# Real-World Shelf Life of Epinephrine Auto-Injectors in Canada: Implications for Renewal Frequency and Patient Burden

**DOI:** 10.3390/jmahp14030054

**Published:** 2026-09-14

**Authors:** John Papastergiou, Mille Vang Lybech, Christoffer Mertz, Anne Danø, Jess Finney, David Grabowski

**Affiliations:** 1 Leslie Dan Faculty of Pharmacy, University of Toronto, Toronto, ON M5S 3M2, Canada; 2 School of Pharmacy, University of Waterloo, Kitchener, ON N2G 1C5, Canada; 3 Shoppers Drug Mart, Toronto, ON M4L 3B5, Canada; 4 ALK-Abelló, 2970 Hørsholm, Denmark; mille.vanglybech@alk.net; 5 Eviproseon, 4320 Lejre, Denmark; 6 Inspiria, 2960 Rungsted Kyst, Denmark; 7 Branding Science Group, London SW18 1DE, UK; jess.finney@branding-science.com; 8 ALK-Abelló Pharmaceuticals Inc., Mississauga, ON L4Z 2H6, Canada; david.grabowski@alk.net

**Keywords:** epinephrine auto-injectors, anaphylaxis treatment, shelf life, disposal practices, expiry, pharmacy inventory management

## Abstract

Although patients in Canada at risk of anaphylaxis are recommended to maintain access to two in-date epinephrine auto-injectors (EAIs) at all times, underuse during emergencies remains common. Multiple factors contribute to this gap, but the impact of real-world shelf life has been understudied. This study examines the remaining in-pharmacy shelf life of EAIs in Canada and considers its potential implications for patient burden, renewal frequency, and anaphylaxis preparedness. In this cross-sectional study, 50 licensed Canadian pharmacists from chain, independent, grocery/mass merchandise, and hospital pharmacies completed an online questionnaire and assessed EAI stock. Participating pharmacists verified all EAI stock on site, including batch numbers and expiry dates, and reported patient disposal practices. Of the 50 participating pharmacists, 49 reported 411 EAI devices across 98 batches. The mean in-pharmacy shelf life at dispensing was 12.8 months, with 47.4% of devices having 12 months or less remaining shelf life. Disposal practices varied and expiry was the most cited reason for disposal. Shortened in-pharmacy shelf life may increase renewal frequency, cost, waste, and the risk that patients carry expired devices or no device during anaphylaxis. Longer-shelf-life epinephrine treatment options or improved distribution chain practices may help reduce patient burden and enhance anaphylaxis preparedness in Canada.

## 1. Introduction

Anaphylaxis is a severe, potentially life-threatening allergic reaction that requires immediate treatment. In Canada, epinephrine auto-injectors (EAIs) are the recommended first-line therapy and should be administered as soon as signs or symptoms of anaphylaxis occur [1,2,3]. Current clinical recommendations advise that patients at risk of anaphylaxis have immediate access to at least two, in-date, weight-appropriate doses of EAIs at all times [1,2]. Ensuring consistent access to these devices is therefore a critical component of anaphylaxis management and prevention of adverse outcomes.

The economic burden associated with allergy and anaphylaxis in Canada is substantial. Food allergy alone has been estimated to incur annual costs of $41.9 billion Canadian dollars (CAD), including $2.8 billion CAD in direct healthcare expenditures. Indirect costs, primarily productivity loss, amounted to an estimated $34.4 billion CAD. Beyond acute care costs, this burden is influenced by chronic management challenges, including the ongoing procurement and replacement of EAIs [4].

Despite clear guidelines, real-world underuse of EAIs during anaphylactic reactions remains an issue, even among patients with a history of anaphylaxis, contributing to increased morbidity and healthcare costs [5,6,7,8,9,10,11]. For example, an analysis of data from the Cross-Canada Anaphylaxis REgistry (C-CARE) reported that EAI use in the prehospital setting occurred in only 31% of patients presenting with anaphylaxis [12]. A separate analysis of the C-CARE, using a subset of cases, found that only 41% of pediatric patients with immediate access to an EAI used it before hospital arrival [13]. While underuse is multifactorial, these findings highlight persistent gaps between guideline recommendations and real-world practice.

A range of individual- and system-level barriers contribute to sub-optimal EAI use and availability, including fear of needle-related risks, device complexity and cost, lack of proper training, and portability challenges [5,9,10,14,15,16,17]. Consistent carriage of EAIs is notably poor. A Canadian survey found that 57% of individuals at risk of anaphylaxis did not consistently carry their EAI [18]. Similar patterns have been observed internationally. A large US survey of 2000 adults and children who had filled at least one prescription for an EAI reported that only 50% carried their device at all times during the previous week [19]. Furthermore, prescription adherence is sub-optimal; in a Manitoba primary care cohort, including 1212 adults, only 70% of EAI prescriptions were filled [10,20]. Comparable challenges have been reported elsewhere. For example, a Danish study investigating the EAI collection rate of patients who had received emergency care for anaphylaxis and subsequently been through diagnostic work up, found an overall retrieval rate of 76%, with the highest rate observed among patients who had experienced severe anaphylaxis [6]. Together these studies suggest that even within well-resourced healthcare systems, structural and behavioral barriers limit optimal EAI access.

An often-overlooked determinant of EAI access and adherence is the remaining shelf life of available devices at the time of dispensing. At manufacturing release, the two EAIs available in Canada are approved with labeled shelf lives of 19 months (150 mcg dose) and 24 months (300 mcg dose) [21]. However, the real-life EAI shelf life available to patients at dispensing will always be shorter due to distribution timelines, wholesaler and pharmacy storage, and inventory turnover. This is supported by a study characterizing real-world shelf life of EAIs in Denmark, Finland, Sweden, and Norway, where the mean remaining in-pharmacy shelf life ranged from 4.3 to 11.4 months depending on dose and country [22]. As a result, patients may receive devices with substantially reduced remaining shelf lives, necessitating more frequent prescription renewals and increasing financial, logistical, and adherence burdens. These challenges may be particularly salient for families of pediatric patients, who are often required to maintain multiple devices across home, school, and childcare settings.

In Canada these access challenges have been further exacerbated by recurrent EAI supply shortages, during which availability to patients have been constrained [23,24]. At the time of writing, only a single EAI brand is available on the Canadian market, amplifying the potential impact of limited remaining in-pharmacy shelf life. Despite these concerns, empirical data describing the real-world shelf life of EAIs at the point of dispensing are scarce, and little is known about how pharmacists manage near-expiry devices, including disposal practices.

The objective of this study was to characterize the remaining in-pharmacy shelf life of EAIs available in Canada—defined as the shelf life of EAIs available in pharmacies prior to dispensing—and to explore pharmacist-reported disposal practices. It further considers the potential implications of in-pharmacy shelf life for patient burden, renewal frequency, and anaphylaxis preparedness. To our knowledge, this is the first study to examine in-pharmacy stock shelf life of EAIs and disposal practices in Canada.

## 2. Materials and Methods

### 2.1. Data Collection

This cross-sectional study was conducted using an online survey to assess the in-pharmacy shelf life of EAIs and pharmacist-reported disposal practices in Canadian pharmacies. The survey targeted licensed pharmacists practicing in a range of pharmacy settings, including chain, independent, grocery/mass merchandise, and hospital pharmacies.

Participants were recruited through Sermo, a third-party quantitative panel provider. Potential participants were invited via direct email outreach and through Sermo’s online panel. To be eligible, respondents were required to:

1. Be licensed pharmacists practicing in Canada;
2. Work in a pharmacy that had EAIs in stock at the time of the survey;
3. Be able to directly verify EAI batch numbers and expiry dates from on-site inventory.

To prevent clustering by site, only one pharmacist per pharmacy location was permitted to complete the survey. Selection of participants was not conducted through fully random sampling. Instead, recruitment aimed to ensure broad geographical diversity, with participation across provinces broadly reflecting their population size, while also balancing rural and urban settings and including pharmacies from multiple practice settings. A total of 50 pharmacists participated in and completed the survey, and all completed surveys were included in the analysis. Participants received an honorarium of $39 CAD upon survey completion, consistent with market research norms for professional surveys.

The survey instrument included questions on EAI inventory and patient disposal practices. Participants were asked to identify the expiry date of all EAIs currently in stock. This approach was selected instead of prospectively recording dispensed EAIs at pharmacies to enable a feasible and broad assessment across pharmacies, geographical regions, and practice settings.

Participants were also asked to report how patients typically dispose of their EAIs after dispensing. Responses were based on the pharmacists’ knowledge and experience at their respective pharmacies; no patient questionnaire or database was used. For batches disposed of by the pharmacy, respondents were asked to list the top three reasons for disposal from the following options: expired device, used device, temperature concerns, quality concerns, device fault, or other.

### 2.2. Data Analysis

For each reported EAI, expiry dates were converted into remaining in-pharmacy shelf life at the time of survey completion. Remaining shelf life was calculated as the number of days from survey completion up to and including the expiry month and then converted into standard months of 30.44 days. Two batch-level observations were excluded from the dataset due to implausibly high reported stock levels of (300 and 54 devices for 300 µg and 150 µg EAIs, respectively).

Analyses were descriptive in nature and not intended to formally test hypotheses. Summary statistics, including counts, proportions, medians, and ranges, were used, as appropriate, to characterize the distribution of in-pharmacy shelf life at the time of dispensing and pharmacist-reported disposal practices. All analyses were conducted at the pharmacy or product level, and no inferential statistical testing was performed.

## 3. Results

Of the 50 pharmacists who completed the online survey between 24 June 2025, and 14 July 2025, 28 (56%) worked in chain pharmacies, 11 (22%) in independent pharmacies, 6 (12%) in grocery or mass merchandise pharmacies, and 5 (10%) in hospital pharmacies. Pharmacists from eight different regions completed the survey, with the highest proportions of participants from Ontario (50%), Quebec (18%), and British Columbia (14%), as shown in Table 1. Overall, provincial participation broadly reflected the population distribution across Canada, although Ontario was overrepresented and Alberta underrepresented relative to their respective population sizes. No pharmacists from Prince Edward Island or Manitoba completed the survey.

Forty-nine pharmacies had at least one EAI in stock. The distribution by pharmacy type, location, and province is shown in Table 1.

Across all pharmacies, the mean stock of EAIs (combined doses) was 8.4 devices, ranging from 0 to 20 per pharmacy (Table 2). On average, pharmacies stocked 5.3 devices of the 300 mcg dose compared to 3.1 devices of the 150 mcg dose.

The mean in-pharmacy shelf life of EAIs for both 150 mcg and 300 mcg combined was 12.8 months (median: 12.2 months; range: 0.9–23.9 months) based on a total of 411 devices from 98 batches, see Table 2. Shelf life varied by location, with rural pharmacies showing substantially longer mean shelf life compared to urban/suburban pharmacies (Table 2).

**Figure 1:**
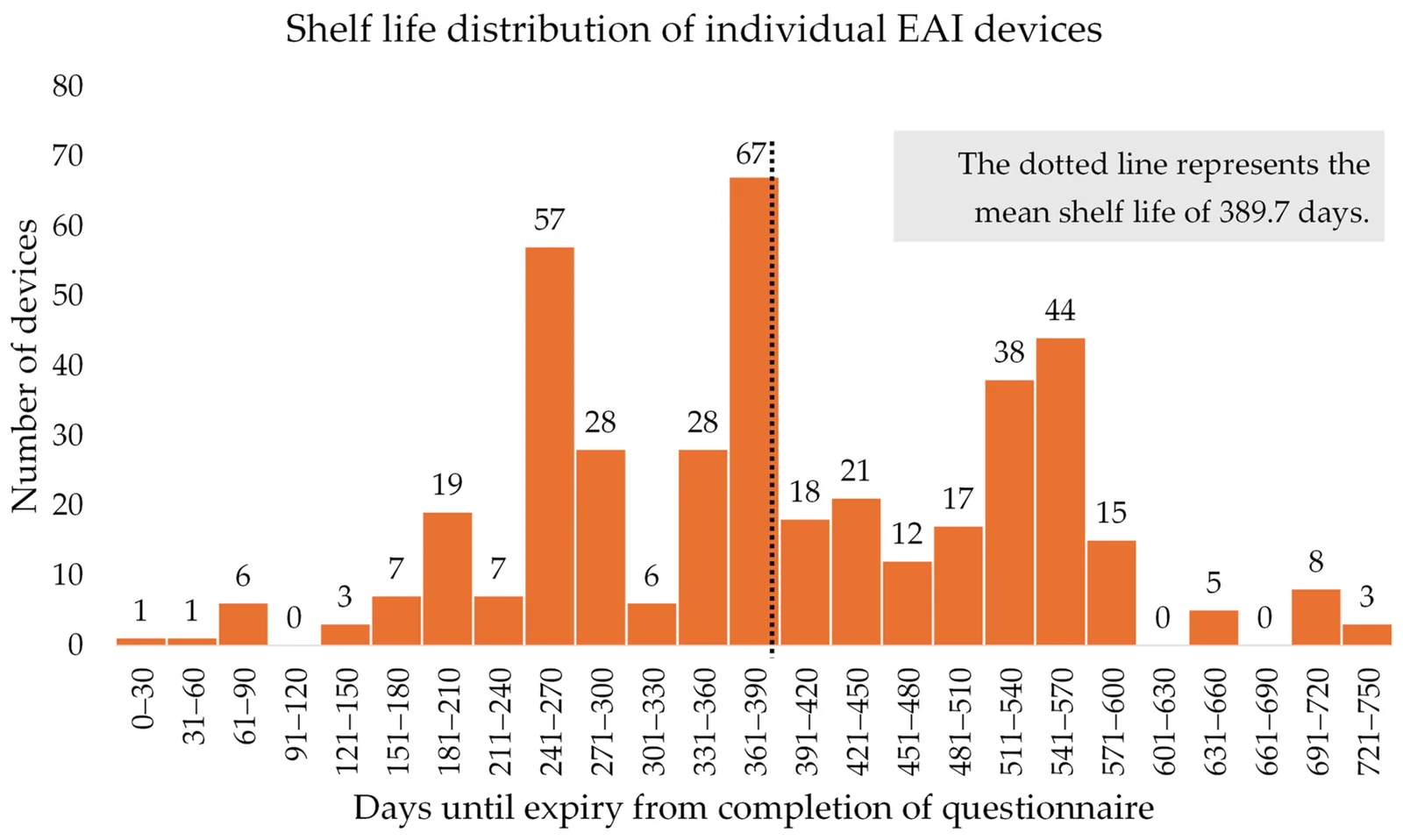
Remaining shelf life of EAIs by devices in stock across pharmacies.

Remaining shelf life of individual devices is illustrated in Figure 1. Of the 411 devices identified, 195 (47.4%) had a shelf life of one year or less. Shelf life of devices was unevenly distributed, with peaks at 241–270, 361–390, and 511–570 days, corresponding to approximately 8–9, 13–14, and 17–19 months. Very few devices had less than 3 months (1.9%) or more than 21 months (3.9%) remaining. The proportion of batches around 12 months indicates that a significant proportion of EAIs will require replacement within one year of dispensing, potentially increasing refill frequency and cost burden.

All 50 respondents completed the survey questions on disposal. Pharmacists reported that patient disposal practices were distributed as follows: returning EAIs to pharmacies (32%), disposal via sharps bin (32%), and disposal by the patient via unspecified methods (33%). Among EAIs disposed of by pharmacists, 100% cited expiry as a top-three reason for disposal, 91% cited prior use, and 67% cited patient device concerns. On average, pharmacists estimated that 16% of patients expressed concern about self-injecting or injecting a child.

## 4. Discussion

Clinical recommendations for anaphylaxis recommend that patients maintain immediate access to two EAIs at all times [1,2]. Although these devices are approved with a labeled shelf life of up to 24 months depending on dose strength, the in-pharmacy shelf life at dispensing is considerably shorter [21]. In this study, the mean in-pharmacy shelf life at dispensing was 12.8 months, and 47.4% of devices had one year or less remaining. This gap between labeled and real-world shelf life illustrates a practical challenge: patients may need to replace devices more frequently than expected. Such shortened shelf life drives an increased renewal frequency for patients, compared to an optimal scenario. Based on our findings, patients may need to refill two EAIs approximately once a year to adhere to current clinical recommendations [1,2]. This requirement imposes additional cost and inconvenience for patients and caregivers, particularly those managing pediatric anaphylaxis.

There is limited data on adherence to timely replacement of EAIs, although lack of adherence to carriage recommendations is well established in Canada [13,18,20]. The relatively short in-pharmacy shelf life observed in this study may represent an additional barrier to maintaining access to in-date EAIs, although further research is needed to better understand its impact on patient renewal behavior and adherence. Additionally, shorter shelf life increases the likelihood of carrying expired devices or having no device available during an emergency. Both of these scenarios are associated with poor outcomes and, in some cases, fatality [25,26,27,28]. The extent to which patients rely on expired devices during real-world emergencies remains unclear, although such use is clearly inadvisable [1,2,21,25,26,27].

If in-pharmacy shelf life contributes to reduced carriage and timely replacement, the implications extend beyond individual patients to healthcare utilization and societal costs [4]. Addressing these barriers could reduce emergency visits and overall expenditure [4,29]. This is particularly relevant if the current paradigm of activating emergency medical services (EMS) for all anaphylaxis cases shifts toward selective at-home management for patients without risk factors and with rapid symptom resolution after prompt EAI administration [1,30]. Such a shift has been estimated to be cost-effective in Canada; however, its potential impact on EMS utilization would be limited unless carriage and timely use of EAIs improve [29].

Disposal practices further compound this challenge. EAIs are frequently discarded due to expiry or device concerns, adding to renewal frequency already strained by periodic shortages [23,24]. These factors highlight the need for innovations in emergency treatment, such as formulations with extended shelf life or alternatives that do not rely on strict temperature control [10]. Beyond the patient perspective, shortened in-pharmacy shelf life also creates inefficiencies across the wider distribution chain. Because devices must move through manufacturers, distributors, wholesalers, pharmacies, hospitals, and emergency services within a narrower usable window, reduced shelf life increases inventory pressure, accelerates waste, and raises costs at multiple points before the device even reaches patients. Longer-shelf-life products could therefore reduce waste, alleviate distribution inefficiencies, improve adherence, and potentially increase adoption of EAI stocking in public spaces.

This study has several limitations. Interpretation of the mean and median in-pharmacy shelf life estimates are limited by the inclusion of both 150 mcg and 300 mcg EAI devices, which have different labeled shelf lives and are intended for different patient populations. Differences in the distribution of these devices within the sample may therefore influence the overall estimates and limit direct inferences to either pediatric or adult patients.

The relatively small sample size, the absence of participating pharmacists from Manitoba and Prince Edward Island, and the short data collection period limit the generalizability of the findings. Repeating the study would help determine whether the findings reflect a persistent trend. Nonetheless, concerns regarding EAI shelf life have been reported by both patients and healthcare professionals, and similar real-world shelf life estimates have been observed in Denmark, Finland, Sweden, and Norway [22].

Additionally, the study assessed pharmacy stock and in-pharmacy shelf life rather than patient purchasing behavior, dispensing practices, or adherence. In practice, patients may receive devices with shorter shelf life than the average observed in this study, as pharmacies typically dispense stock with the earliest expiry first. A prospective study evaluating dispensing practice and shelf life at the time of dispensing would complement these findings by providing a more direct estimate of the shelf life available to patients. Future research is also needed to better understand the relationship between EAI shelf life, patient adherence, and clinical outcomes.

## 5. Conclusions

The in-pharmacy shelf life of EAIs available at Canadian pharmacies is substantially shorter than the labeled shelf life at manufacturing release, potentially increasing treatment costs, medication waste, and patient burden. Improving distribution chain practices and access to longer-shelf-life epinephrine treatment options may help reduce these burdens and enhance anaphylaxis preparedness in Canada. Further research is needed to better understand the relationship between shelf life, patient adherence and clinical outcomes.

## Figures and Tables

**Table 1:** Distribution by pharmacy type, location, and province.

**Type of Pharmacy**	**Urban/Suburban**	**Rural**	**Total**
Chain pharmacy, n (%)	23 (82.1)	5 (17.9)	28
Independent pharmacy, n (%)	9 (81.8)	2 (18.2)	11
Grocery/mass merchandise pharmacy, n (%)	6 (100)	0 (0)	6
Hospital pharmacy, n (%)	4 (80)	1 (20)	5
**Province**			**n (%)**
Ontario			25 (50.0%)
Quebec			9 (18.0%)
British Columbia			7 (14.0%)
Nova Scotia			3 (6.0%)
Alberta			3 (6.0%)
Saskatchewan			1 (2.0%)
New Brunswick			1 (2.0%)
Newfoundland and Labrador			1 (2.0%)

Note: n refers to the number of observations.

**Table 2:** Stock levels and shelf lives of EAIs at pharmacies.

**Device Stock Levels**	**Mean**	**Median**	**Range**
300 mcg device ^a^ (n = 260)	5.3	5	0–20
150 mcg device ^b^ (n = 151)	3.1	3	0–10
Total device stocks (n = 411)	8.4	8	0–20
**Device Shelf Lives at Stock**			
Urban or suburban (n = 366), months	12.6	12.1	0.9–23.9
Rural (n = 45), months	14.3	16.2	1.1–19.3
Total (n = 411), months	12.8	12.2	0.9–23.9

^a^ 48 pharmacies stocked 300 mcg; ^b^ 47 pharmacies stocked 150 mcg. Note: n refers to the number of observations.

## Data Availability

The datasets generated and analyzed during the current study are proprietary to ALK-Abelló and are not publicly available due to commercial restrictions. Summary results are provided in the manuscript. Anonymized or aggregated extracts that support the findings may be made available on request to the corresponding author.
